# A novel *ex vivo* lung cancer model based on bioengineered rat lungs

**DOI:** 10.3389/fbioe.2023.1179830

**Published:** 2023-06-26

**Authors:** Satoshi Mizoguchi, Tomoshi Tsuchiya, Ryoichiro Doi, Tomohiro Obata, Mayumi Iwatake, Shintaro Hashimoto, Hirotaka Matsumoto, Hiroshi Yukawa, Hiroko Hayashi, Tao-Sheng Li, Kazuko Yamamoto, Keitaro Matsumoto, Takuro Miyazaki, Koichi Tomoshige, Takeshi Nagayasu

**Affiliations:** ^1^ Department of Surgical Oncology, Nagasaki University Graduate School of Biomedical Sciences, Nagasaki, Japan; ^2^ Department of Thoracic Surgery, Faculty of Medicine, Academic Assembly, University of Toyama, Toyama, Japan; ^3^ School of Information and Data Sciences, Nagasaki University, Nagasaki, Japan; ^4^ Institute of Nano-Life-Systems, Institutes of Innovation for Future Society, Nagoya University, Nagoya, Japan; ^5^ Department of Pathology, Nagasaki University Graduate School of Biomedical Sciences, Nagasaki, Japan; ^6^ Department of Stem Cell Biology, Atomic Bomb Disease Institute, Nagasaki University, Nagasaki, Japan; ^7^ Department of Respiratory Medicine, Nagasaki University Graduate School of Biomedical Sciences, Nagasaki, Japan

**Keywords:** decellularization, recellularization, 3D culture, cancer model, bioengineering

## Abstract

**Introduction:** Two-dimensional cell cultures have contributed substantially to lung cancer research, but 3D cultures are gaining attention as a new, more efficient, and effective research model. A model reproducing the 3D characteristics and tumor microenvironment of the lungs *in vivo*, including the co-existence of healthy alveolar cells with lung cancer cells, is ideal. Here, we describe the creation of a successful *ex vivo* lung cancer model based on bioengineered lungs formed by decellularization and recellularization.

**Methods:** Human cancer cells were directly implanted into a bioengineered rat lung, which was created with a decellularized rat lung scaffold reseeded with epithelial cells, endothelial cells and adipose-derived stem cells. Four human lung cancer cell lines (A549, PC-9, H1299, and PC-6) were applied to demonstrate forming cancer nodules on recellularized lungs and histopathological assessment were made among these models. MUC-1 expression analysis, RNA-seq analysis and drug response test were performed to demonstrate the superiority of this cancer model.

**Results:** The morphology and MUC-1 expression of the model were like those of lung cancer *in vivo*. RNA sequencing revealed an elevated expression of genes related to epithelial-mesenchymal transition, hypoxia, and TNF-α signaling via NF-κB; but suppression of cell cycle-related genes including E2F. Drug response assays showed that gefitinib suppressed PC-9 cell proliferation equally well in the 3D lung cancer model as in 2D culture dishes, albeit over a smaller volume of cells, suggesting that fluctuations in gefitinib resistance genes such as JUN may affect drug sensitivity.

**Conclusions:** A novel *ex vivo* lung cancer model was closely reproduced the 3D structure and microenvironment of the actual lungs, highlighting its possible use as a platform for lung cancer research and pathophysiological studies.

## 1 Introduction

Lung cancer is the leading cause of cancer-related deaths worldwide, and its 5-year survival rate is only 19% (Siegel et al., 2021). Epidermal growth factor receptor thyroxin kinase inhibitors (EGFR-TKIs) or anaplastic lymphoma kinase TKIs have improved lung cancer treatment (Mitsudomi et al., 2010; Solomon et al., 2014), and immunotherapy using immune checkpoint inhibitors shows great potential (Reck et al., 2016; 2019). However, these therapies can benefit only a limited proportion of patients with lung cancer, and new drugs are needed. Unfortunately, many newly developed drugs, which have succeeded in preclinical studies, have failed to show the desired effect in phase II or phase III clinical trials (Dimasi et al., 2013; Hay et al., 2014).

One reason for this failure is the absence of an accurate evaluation platform for newly developed oncologic agents. For one, two-dimensional (2D) cultures of cancer cells in a plate or flask, which is what most anticancer drugs are studied in, are not identical to the same cells cultivated *in vivo* on a three-dimensional (3D) structure (Griffith and Swartz, 2006; Yamada and Cukierman, 2007; Kimlin et al., 2013). Crucially, 3D cultured cells co-exist within a microenvironment that mimics native organ tissue, and this is critical for the development of cellular characteristics. The tumor microenvironment plays an important role in cell proliferation, invasion, differentiation, and function, directly affecting drug responses. Cancer cells co-cultured with cancer extracellular matrix (ECM) derived via decellularization exhibit drug resistance. Specifically, TGF-β released by cancer cells binds to chondroitin sulfate in the ECM, which enhances TGF-β signaling, facilitates the epithelial-mesenchymal transition (EMT), and induces the ABCB1 drug transporter (Hoshiba and Tanaka, 2016). The different NF-κB expression between lung cells cultured in 3D vs. 2D mode have been related to gefitinib resistance (Sakuma et al., 2012). Further differences may arise due to cell-to-cell or cell-to-ECM crosstalk. Thus, 3D lung cancer models comprising non-cancerous alveolar cells and lung cancer cells could dramatically improve cancer research.

Various 3D models for the analysis of cancer cell behavior, such as lung-on-a-chip and organoids, are already available. Lung-on-a-chip models comprise layers of alveolar epithelial cells and microvascular endothelial cells seeded above and below a basement membrane made of polydimethylsiloxane, a versatile biomaterial (Huh, 2015; Konar et al., 2016). A next-generation lung-on-a-chip model that replaces polydimethylsiloxane with collagen and elastin, the main components of the ECM, has recently been described (Zamprogno et al., 2021). Although this model offers improved stretchability and biodegradation, both of which are important for tissue remodeling, the conformation of its air-blood barrier remains planar or cylindrical, which cannot reproduce a complex 3D structure containing different cells and cancerous nodules. Organoids are commonly cultured at the air-liquid interface using a hydrogel containing ECM, such as matrigel (Nadkarni et al., 2016; Barkauskas et al., 2017). There have been reports of lung cancer models using organoid technology, but most are actually 3D spherical structures consisting only of cancer cells (Kim et al., 2019). Some organoids have been produced by co-culturing cancer-associated fibroblasts with lung cancer cells (Nakamura et al., 2019), but there are no reports of lung cancer organoids established in coexistence with healthy alveolar epithelial cells or lung stromal cells, as happens *in vivo*. Although 3D cultured organoids offer several advantages over 2D options, they still lack a stretchable and complex 3D structure, do not allow cell-cell interaction with non-cancerous cells, and take a long time to form, usually over 2 weeks (Barkauskas et al., 2017).

Decellularization is a regeneration procedure first reported in the 1980s that yields acellular scaffolds or ECM after removal of the cell component. Since 1994, the technique has been applied to produce artificial skin or biological valves. Bioengineered artificial organs created from acellular organ scaffolds are attracting increasing attention to solve the severe donor shortage in transplantation therapy (Ott et al., 2010; Petersen et al., 2010; Song et al., 2013).

Regenerated lungs made of an acellular scaffold preserve the original 3D architecture and are rich in ECM. Repopulation of these lungs with new cells from the intended recipient could more accurately reproduce the lungs *in vivo*. The first bioengineered lungs were produced using detergents or enzymes to remove cellular material, followed by cell repopulation via the vasculature and airways. When transplanted, the model allowed air exchange through ventilation, albeit for a short time only (Ott et al., 2010; Petersen et al., 2010). Nevertheless, *ex vivo* bioengineered lungs have a key advantage, namely, that their structure and cell population are like those in actual lungs. Here, we hypothesized that the implantation of cancer cells in bioengineered lungs could mimic the cancer niche and establish a novel 3D model of human lung cancer *ex vivo* to facilitate the study of cancer biology or predict drug responses.

## 2 Materials and methods

### 2.1 Harvesting and decellularization of rat lungs

Rat lungs were obtained from 8 to 10-week-old male Fischer 344 rats (CLEA Japan). All animal experiments were performed following approval by the Nagasaki University Institutional Animal Care and Use Committee, and all animal manipulation complied with the Guide for the Care and Use of Laboratory Animals (approval no. 190918-1-6). To harvest the organs, rats were first anesthetized by intraperitoneal injection of ketamine and xylazine and, following a tracheotomy, intubated with a 16G catheter for ventilation. A transverse incision was made in the abdomen, and an anterior wall of the thorax was removed. After heparinization, the apex of the heart was excised and PBS containing heparin (100 U/mL) and sodium nitroprusside (10 μg/mL) were perfused via the pulmonary artery (PA) to remove blood. The lungs were carefully dissected with the trachea and heart and removed *en bloc*. Decellularization of the lungs was conducted by perfusion through the PA, after the trachea and PA/pulmonary vein (PV) of freshly resected lungs were cannulated (Doi et al., 2017; Obata et al., 2019). A sequential perfusion of PBS with calcium/magnesium (PBS+) and PBS+ with 0.0035% Triton-X was performed. Then, the lungs were successively perfused with increasing concentrations of SDS (0.01%, 0.05%, and 0.1%), followed by 1% Triton-X in deionized water. Subsequently, the vasculature of the lungs was rinsed with 2 L of PBS and perfused with PBS containing penicillin (100 U/mL), streptomycin (100 μg/mL), gentamycin (10 μg/mL), and amphotericin-B (2.5 μg/mL), and stored at 4°C for future use.

### 2.2 Isolation of rat primary epithelial cells and adipose-derived stem cells

The lungs of 3-4-week-old male Fischer 344 rats were harvested as described above. After removal from the thorax, the lungs were rinsed several times with cold PBS via the trachea, injected first with elastase (4.5 U/mL; Worthington Biochemical Corp.) in Dulbecco’s modified Eagle medium/nutrient mixture F-12 (DMEM/F-12) containing 2.5% HEPES and DNase I (0.01 mg/mL; Sigma-Aldrich) and then with 1% low-melting-point agarose, and placed immediately on ice. The heart was removed from the surrounding tissue, and the remaining tissue, including the lungs, was incubated in a shaking water bath at 37°C and 100 rpm for 45 min. The trachea and proximal bronchus were removed, and the lungs were minced into small pieces of less than 1 mm^3^ with sterilized fine scissors. After another 15-min incubation at 37°C and 100 rpm with a new elastase solution, DMEM/F-12 containing 50% FBS was added, the minced lungs were placed on ice for 5 min, sequentially filtered through 100-μm/70-μm cell strainers, and centrifuged for 5 min at 300 × *g*. Primary pulmonary cells were used immediately after isolation.

Adipose-derived stem cells (ADSC) were isolated, as previously described (Zuk et al., 2001). Briefly, inguinal adipose tissue from young male Fischer 344 rats (6–7-weeks old) was cut into pieces of less than 5 mm^3^ with sterilized scissors. The minced tissue was digested with collagenase (Celase; Cytori Therapeutics) for 30 min in a shaking bath at 37°C and then centrifuged at 400 × g for 5 min. To inactivate collagenase, 5% bovine serum albumin in PBS was added. After several cycles of shaking and centrifugation, the mixture was filtered successively through 100-μm/40-μm cell strainers. Following a final centrifugation step at 400 × *g* for 5 min, the supernatant was discarded, and the cells were resuspended in ADSC growth medium (Lonza) in a sterile dish. Isolated ADSC were used at passage 2–4 for recellularization.

### 2.3 Recellularization and culture of rat lungs

The decellularized rat lung scaffold was connected to a biomimetic bioreactor as previously described (Petersen et al., 2010; Calle et al., 2011). Isolated rat primary pulmonary cells were reseeded via the airway by gravity, typically with a concentrated suspension containing ∼20 × 10^6^ cells, and incubated in a DMEM/F-12 medium at 37°C and 5% CO_2_. After static overnight incubation, the reseeded lung scaffolds received ∼30 × 10^6^ rat microvascular endothelial cells in suspension by gravity via PA and PV, and were statically incubated for 90 min. The reseeded lungs were started on PA perfusion at 1 mL/min and increased by 1 mL/min every 24 h until it reached 4 mL/min and ventilated through the airways with negative pressure at 1 breath/min. The culture chamber contained DMEM/F-12 and endothelial growth medium (EGM-2; Lonza) in a 1:1 proportion, whereas the breathing chamber contained DMEM/F-12 only.

### 2.4 Quantum dots labeling of human lung cancer cells

Quantum dots (QDs) are inorganic probes comprising CdSe/ZnS-core/shell semiconductor nanocrystals, and a useful low-cytotoxicity tool for fluorescent cell labeling. The transduction of QDs was conducted as previously described (Yukawa et al., 2009). Briefly, A549 and PC-9 cells were incubated in a culture medium containing 2 nM Qdot™ 800 ITK™ Carboxyl Quantum Dots (Invitrogen) for 24 h. After washing with PBS, a suspension of 2.5 × 10^6^ QD-labeled lung cancer cells was injected directly into each part of a decellularized rat lung scaffold using 30G needles. Following overnight perfusion cultivation (1 mL/min), the rat lung scaffold was subjected to IVIS Imaging System, IVIS Lumina K (PerkinElmer). The fluorescence derived from QDs800 in specimen was detected with IVIS Lumina K at the condition of excitation filter: 740 nm ± 10 nm, emission filter: 790 nm ± 15 nm.

### 2.5 Seeding with human lung cancer cells

Human lung cancer cell lines were provided by the Department of 2^nd^ Internal Medicine and the Department of Human Genetics, Atomic Bomb Disease Institute, Nagasaki University. A549 and NCI-H1299 cells were cultured in DMEM/F-12, whereas PC-6 and PC-9 cells were cultured in an RPMI-1640 medium; both media were supplemented with 10% FBS and antibiotics/antimycotics. Re-epithelialized/re-endothelialized rat lungs were incubated for 24 h before lung cancer cell seeding. After trypsinization, a suspension of 2.5 × 10^6^ cells from each cell line was injected locally using a 30G needle. Typically, 3–4 infusions were made for each recellularized set of lungs. After static incubation at 37°C and 5% CO_2_ for 1 h, the culture medium was refreshed and PA perfusion/ventilation was restarted. PA perfusion increased from 1 mL/h to 4 mL/h daily. Following 72-h incubation, the bioengineered lungs were prepared for further analysis.

### 2.6 Histology and immunohistochemistry

Lung samples were fixed for 24 h with 4% paraformaldehyde in PBS, embedded in paraffin, and sectioned into 5-μm-thick slices. The slices were stained with hematoxylin and eosin, Elastica van Gieson, and periodic acid-Schiff stain, according to standard protocols.

For immunohistochemistry, tissue sections were deparaffinized, rehydrated, and rinsed with PBS several times. Antigen retrieval was performed in 10 mM citrate buffer (pH 6) at 121°C for 15 min. Slides for enzyme labeled antibody method were additionally incubated in 3% H_2_O_2_ for endogenous peroxidase inactivation for 30 min at room temperature, then all slides were blocked with 5% normal goat serum in PBS for 1 h, incubated with primary antibodies overnight at 4°C and secondary antibodies for 1 h at room temperature, and finally mounted with 3,3ˊ-diaminobenzidine. Anti-Ki67 (ab16667, 1:1000; Abcam) and anti-MUC1 (ab109185, 1:250; Abcam) were used as primary antibodies.

Slides for bright field were imaged on a Leica DM6000 (Leica Microsystems GmbH) microscope. Immunofluorescent slides were imaged on an Invitrogen EVOS M7000 Imaging System (Thermo Fischer Scientific). For quantitative analysis, 10 high-power views at a ×400 magnification were counted for each sample (*n* = 3) and analyzed using ImageJ software (National Institutes of Health).

### 2.7 Western blot

Proteins from 2D and 3D cell cultures (2 nodules per each sample) were extracted in RIPA buffer containing protease and phosphatase inhibitor cocktails (Nacalai Tesque) according to the manufacturer’s instructions. Proteins were quantified with a Pierce™ BCA Protein Assay Kit (Thermo Fisher Scientific) following measurement in a Thermo Scientific Multiscan FC microplate reader using Scanlt™ software (Thermo Fisher Scientific). Protein samples were separated on 12.5% SDS-polyacrylamide gels (10 μg/lane) and transferred onto a polyvinylidine difluoride membrane. The membranes were blocked with Blocking One-P (Nacalai Tesque) for 30 min, followed by incubation with primary antibodies overnight at 4°C. After washing with TBS containing 0.1% Tween-20, the membranes were incubated with secondary antibodies for 1 h, washed three times with the same buffer, and exposed in a LAS-3000 luminescent image analyzer (GE Healthcare) using the Chemi-Lumi One Super kit (Nacalai Tesque) for protein visualization. Anti-MUC1 (19976-1-AP, 1:1000; Proteintech) was used as the primary antibody and horseradish peroxidase-conjugated goat anti-rabbit IgG H&L (ab205718, 1:10000; Abcam) as secondary antibody.

### 2.8 Cell viability assay

The inhibitory effect of gefitinib on the proliferation of PC-9 cells was evaluated using the Cell Counting Kit-8 (DOJINDO) cell viability assay. Briefly, PC-9 cells were seeded on a 96-well plate at 1 × 10^4^ cells/well. Cells were incubated at 37 C with various concentrations of gefitinib (0, 0.0001, 0.01, 0.1, 1, 5, 10, and 20 μM) for 48 h. The WST-8 reagent was added to each well, and the plate was incubated for 30 min at 37°C. Absorbance of the reduced WST-8 formazan was measured with the Multiscan FC microplate reader using a filter at 450 nm. The assay was performed in quadruplicate for each concentration.

### 2.9 RNA extraction and RNA sequencing

Total RNA was extracted from 2D cultured cells and cancer cells in regenerated 3D lungs using the RNeasy Mini kit (Qiagen) according to the manufacturer’s instructions. Extractions were performed in triplicate for each group. RNA purity was evaluated with a Nano Drop ND-1000 spectrophotometer (Thermo Fisher Scientific). RNA integrity of each sample was verified with an Agilent 2,100 Bioanalyzer using the Agilent RNA 6000 Nano Assay (Agilent Technologies), in which all RNA integrity number values were greater than 8. The samples were sent to Riken Genesis (Japan) for RNA sequencing. An mRNA library was prepared with the TruSeq Stranded mRNA Library Prep kit (Illumina) and paired-end sequencing was performed with NovaSeq 6,000 (Illumina). The quality control of each sample was evaluated with FastQC by Riken Genesis.

### 2.10 RNA-sequencing analysis

Transcript expression in three biological replicates of each test group was quantified using Salmon v.1.4.0 (Patro et al., 2017). Because the sequenced fastq files contained some reads corresponding to rat RNA, we removed such contaminants using the following procedure. First, human and rat transcripts fasta files were downloaded from GENCODE (v.36) and Ensembl (Rnor_6.0), respectively. Second, we merged these fasta files and created an index file using the Salmon index command with the parameter -k 31. Third, we quantified transcript expression using the Salmon quant command with the above index file.

Next, we analyzed the Salmon output files in R. We created the tx2 gene file from the GTF file downloaded from GENCODE (v.36) and converted the Salmon output files into human gene-level expression data using tximport v.1.18.0 (Soneson et al., 2016). We performed differential expression analysis with DESeq2 v.1.30.1 (Love et al., 2014) for the following groups: recellularized vs. 2D cultured, decellularized vs. 2D cultured, and recellularized vs. decellularized. Based on the obtained results, we then performed enrichment analyses. Differentially expressed genes (DEGs) were defined as having an adjusted *p <* 1.0 × 10^−5^ and divided into two gene lists depending on whether the log2 fold change (log2FC) of mean expression was positive or negative. Finally, they were submitted to Metascape (Zhou et al., 2019) and Enrichr (Kuleshov et al., 2016) for gene list enrichment analysis. We also sorted genes according to log2FC and performed gene set enrichment analysis (GSEA) using FGSEA v.1.16.0 against hallmark gene sets downloaded from the Molecular Signature Database (MSigDB).

### 2.11 Statistical analysis

Results are presented as means ± SD. Unpaired two-tailed Student’s *t*-tests were performed to evaluate significant differences between the two groups. *p <* 0.05 was considered significant. All statistical analyses were performed with JMP Pro v15.0.0 software (SAS Institute Inc.).

## 3 Results

### 3.1 Establishment of the *ex vivo* lung cancer model

**FIGURE 1:**
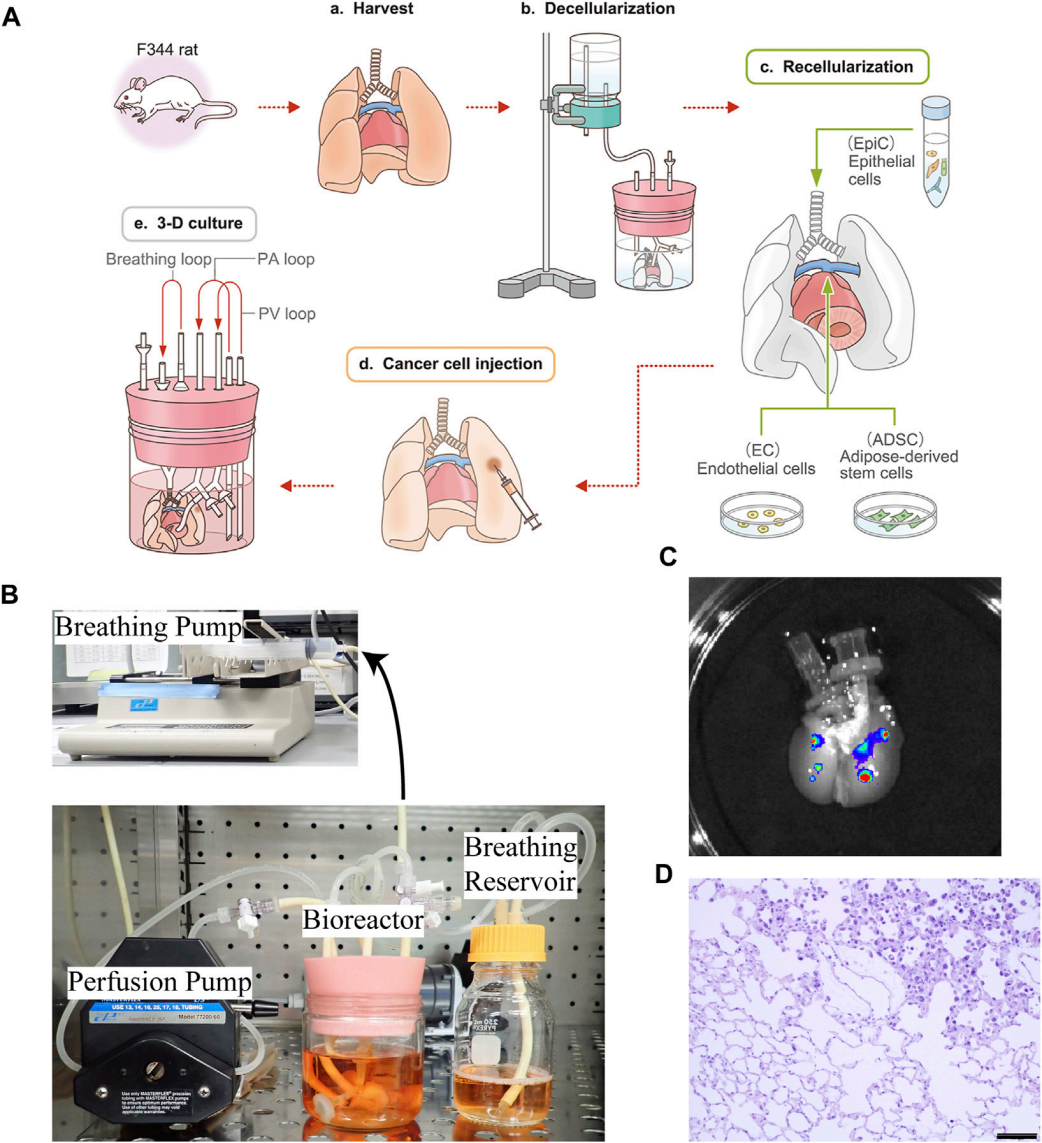
Schematic protocol for creating the *ex vivo* lung cancer model. **(A–a)** Whole lungs and heart were harvested *en bloc* from a Fischer 344 rat. **(A–b)** The harvested lungs were cannulated in the trachea, PA, and PV to perform gravity-perfusion with detergent (SDS) for whole-lung decellularization. Decellularized rat lungs appear white and slightly transparent. **(A–c)** Epithelial cells (EpiCs) isolated from juvenile rats were seeded through the trachea, whereas endothelial cells (ECs) were seeded through the PA with some ADSC to reform the epithelium. **(A–d)** Human cancer suspensions were directly infused into the bioengineered lungs, below the pleura. **(A–e)** *Ex vivo* lung cancer model after 72 h of culturing. **(B)** Actual photograph of a bioreactor and perfusion/ventilation system that enables a whole organ culture of bioengineered rat lungs. **(C)**Macrograph of decellularized lungs exhibiting nodules formed by A549 cells labeled with QDs. **(D)** Histopathological findings of the *ex vivo* lung cancer model: recellularized alveolar cells and disseminated human lung cancer cells co-exist and grow in the decellularized ECM; non-cancerous and cancerous areas are delineated. Scale bar: 100 μm **(D)**.

To create the *ex vivo* lung cancer model, we applied a bioengineering strategy, in which the harvested rat lungs (Figure 1A-a) were cannulated in PA, PV, and trachea, followed by decellularization with SDS via PA perfusion (Figures 1A-b). The epithelial cells were repopulated through the trachea, followed by the addition of endothelial cells and ADSC through PA and PV (Figures 1A-c). The cultured human lung cancer cells were then successfully seeded by local infusion (Figures 1A-d) and, after incubation for 1 h, the regenerated lungs were cultured in a bioreactor using PA perfusion with a pump and negative pressure medium ventilation (Figures 1A-e, B).

To determine whether locally seeded human cancer cells could form nodules in the decellularized rat lungs, we injected QD-labeled A549 cells topically into separate lung lobes of the same decellularized scaffold at 2.5 × 10^6^ cells/injection. The cells were then cultured in the 3D space using PA perfusion and observed by *in vivo* imaging after 1 day, whereby it was confirmed that A549 cells remained at the local injection site and formed clearly visible nodules (Figure 1C).

After culturing for 72 h, coexistence of seeded cancer cells and recellularized non-cancerous cells was observed in the bioengineered lungs by hematoxylin and eosin staining (Figure 1D).

### 3.2 Each cancer cell line regains its own pathological properties within the regenerated lungs

**FIGURE 2:**
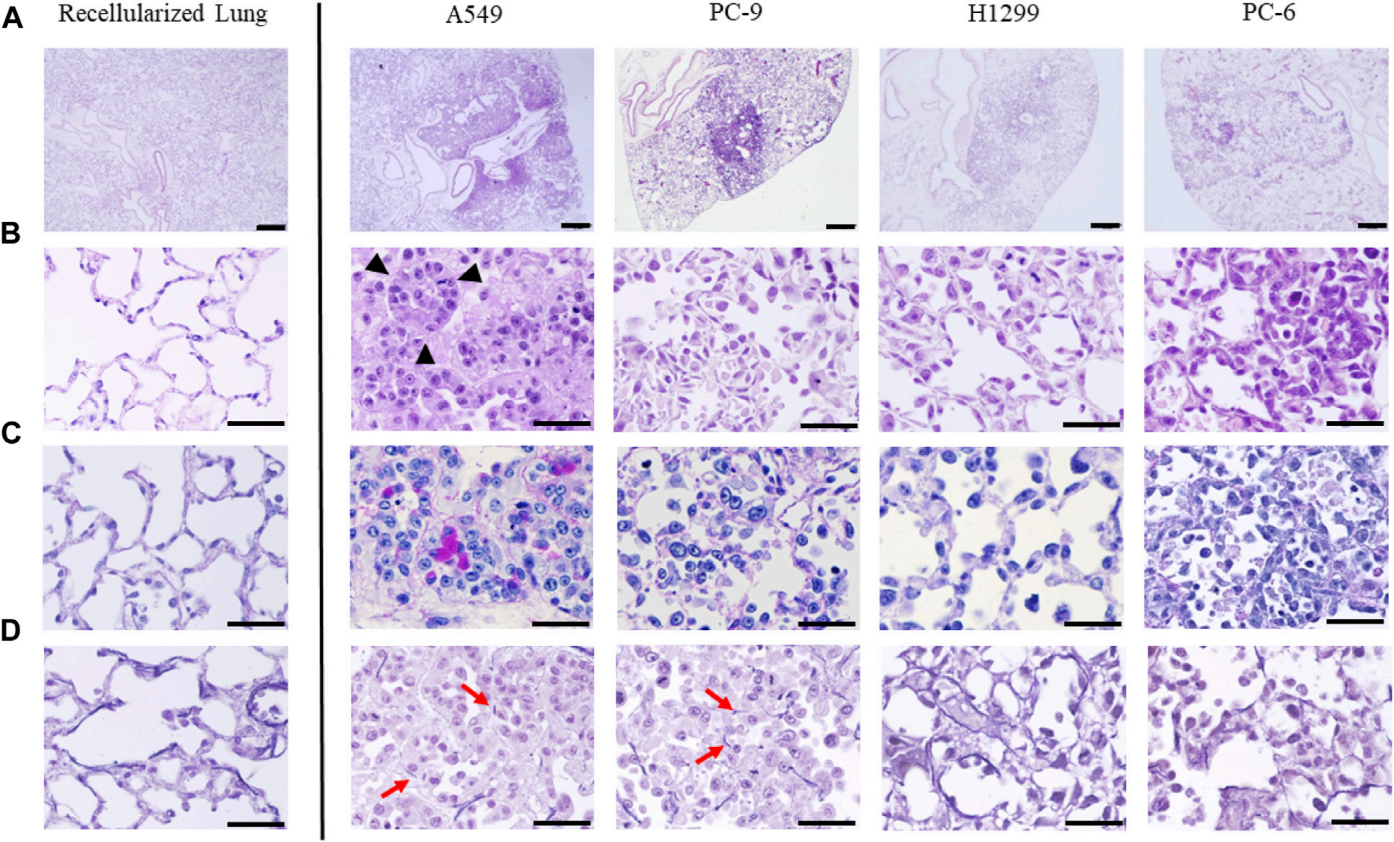
Histopathological evaluation of *ex vivo* lung cancer models generated with four human lung cancer cell lines (A549, PC-9, H1299, and PC-6). Hematoxylin and eosin staining of the control (recellularized lungs) and four cell lines at **(A)** low and **(B)** high magnification. All cell lines formed a nodule on the lungs, and each cell line had a unique appearance. Note the glandular duct structure visible in the strongly magnified image of the model with A549 cells (black arrowheads). **(C)** Periodic acid-Schiff staining showing mucin production by A549 cells. **(D)** Elastica van Gieson staining showing disruption of elastic fibers in the alveolar basement membrane in certain cell lines (red arrows). Scale bar; 500 μm **(A)**, 50 μm **(B–D)**.

To confirm that lung cancer cell lines were seeded in regenerated lungs, we performed histological evaluations (Figures 2A–D). Hematoxylin and eosin staining demonstrated that all cell lines were successfully seeded in the bioengineered lungs as nodules and coexisted with non-cancerous rat lung cells (Figures 2A, B). The histological specificity of the different subtypes of lung cancers was maintained, including adenocarcinoma (A549, PC-9, H1299) and small cell carcinoma (PC-6). A549 cells were densely aggregated and created an acinar structure with mucin, confirmed by periodic acid-Schiff staining as deep purple areas in the cytoplasm (Figure 2C). PC-9 cells also proliferated along the pre-existing alveolar scaffold; however, they lacked acinar structure or mucin production. H1299 cells had spread along alveolar structures similarly to PC-9 cells; they were characterized by typical polygonal shapes and coarse hyperchromatic nuclei. PC-6 cells proliferated along the alveolar structure of the regenerated lungs, but spread outward from the local injection site due to weak adhesion. Elastica van Gieson staining showed that A549 and PC-9 cells had proliferated by destroying the alveolar basement membrane; whereas H1299 and PC-6 cells had proliferated in an epithelial replacement manner without damaging the membrane (Figure 2D).

### 3.3 MUC-1 is overexpressed in the *ex vivo* lung cancer model

**FIGURE 3:**
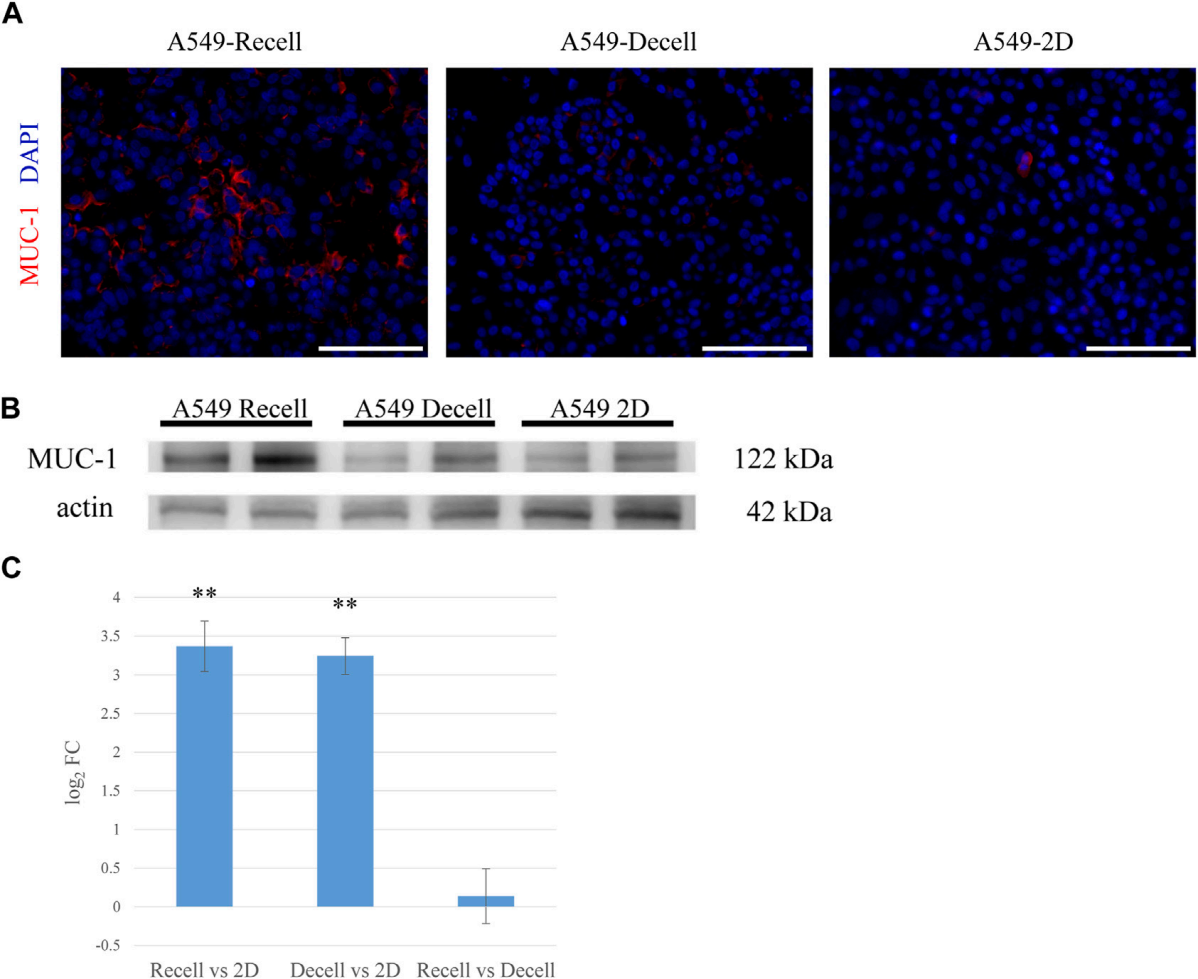
MUC-1 is highly expressed in the *ex vivo* 3D model compared to a 2D culture. **(A)** Fluorescent immunohistological staining showing MUC-1 expression by A549 cells grown in recellularized and decellularized rat lungs and 2D-cultured A549 cells (right, center and left, respectively). **(B)** Western blotting showing MUC-1 expression by A549 cells in recellularized lungs, decellularized lungs, and a 2D dish. **(C)** MUC-1 mRNA expression in A549 cells from recellularized lungs, decellularized lungs, and a 2D dish (n = 3, each). The asterisk indicates the significance of the difference (** *p <* 1.0 × 10^−5^). Scale bar: 100 μm **(A)**.

To determine whether our lung cancer model could reproduce the *in vivo* characteristics of lung cancer, we focused on mucin 1 (MUC-1), a protein associated with lung carcinoma. MUC-1 is expressed by both healthy alveolar epithelial cells and various solid tumors. Its elevated expression by cancer cells is associated with increased proliferation, invasion, and inhibition of apoptosis. In our A549 model, MUC-1 was markedly overexpressed with depolarized expression pattern, reminiscent of *in vivo* lung adenocarcinoma (Figure 3A). Western blotting demonstrated that MUC-1 was significantly more expressed by A549 cells cultured on the recellularized lungs than A549 cells grown as 2D layers in dishes or in decellularized lungs without recellularization (Figure 3B). Finally, RNA-sequencing analysis revealed that MUC-1 mRNA was significantly upregulated in A549 cells cultured in the 3D scaffold with or without recellularization (Figure 3C).

### 3.4 Gene expression profiles revealed by metascape and enrichr analysis

To investigate the differences in gene expression between the 3D lung cancer model and 2D cultures, we performed a transcriptome analysis of total RNA extracted from A549 cells (*n* = 3) grown in recellularized lungs or in culture dishes. RNA sequencing results were submitted to the functional enrichment analysis of DEGs.

**FIGURE 4:**
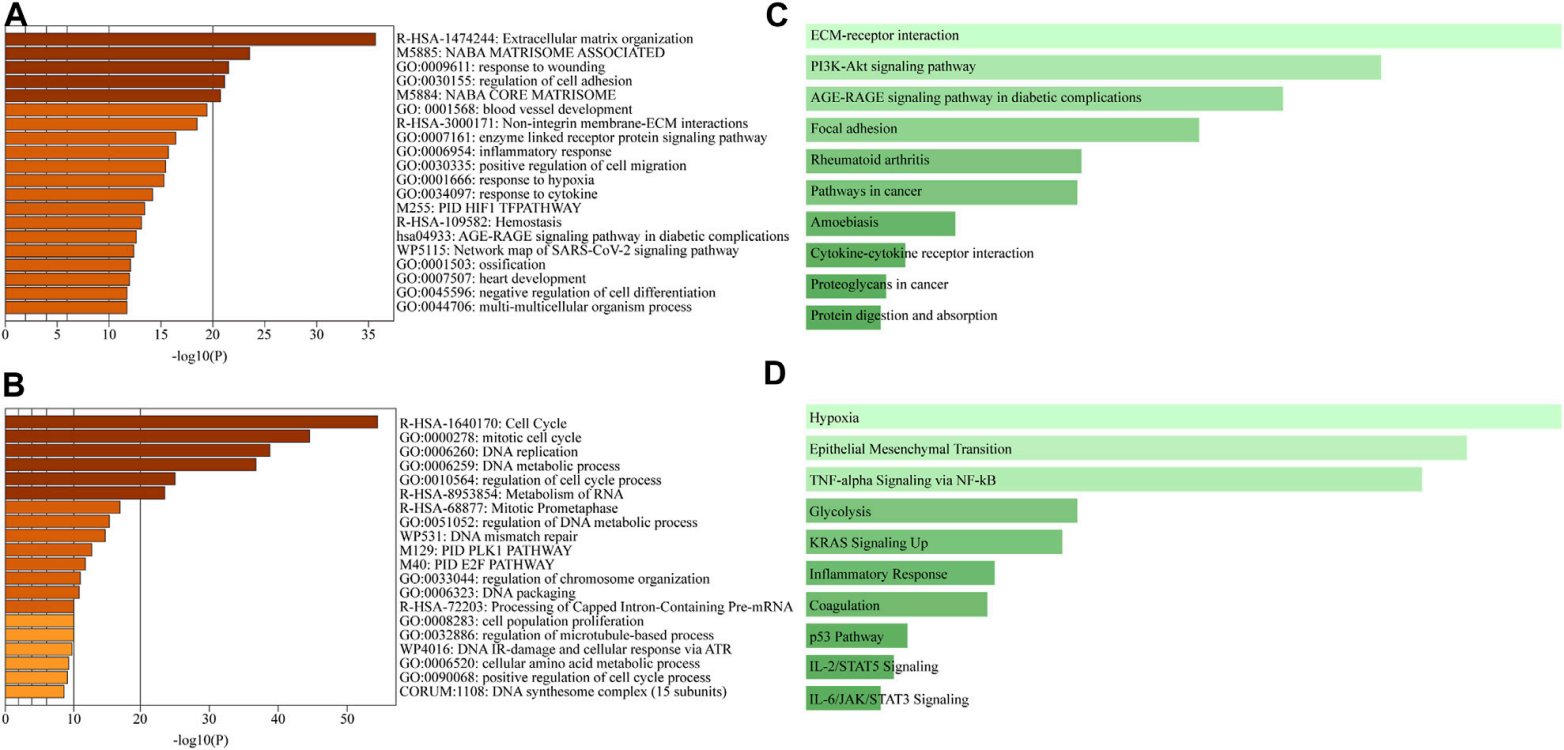
Comprehensive GSEA and pathway enrichment. **(A, B)** Bar graphs obtained through Metascape showing the top 20 enriched terms among **(A)** upregulated or **(B)** downregulated DEGs of A549 cells in the *ex vivo* lung cancer model compared to a 2D culture. **(C, D)** Bar graphs obtained through Enrichr showing pathway enrichment analysis across upregulated DEGs based on **(C)** the KEGG pathway or **(D)** the MSigDB hallmark gene sets.

Metascape integrates large data sets for subsequent comprehensive gene annotation and analysis (Zhou et al., 2019). Metascape identified ECM organization as the most highly upregulated gene family in the recellularized group, followed by structural genes involved in cell adhesion, angiogenesis, and collagen formation (Figure 4A). In contrast, genes related to the cell cycle, such as cell cycle control and regulation, DNA replication, and the PLK1 and E2F pathways, were significantly downregulated in the recellularized group (Figure 4B). Next, we performed GSEA using Enrichr (https://maayanlab.cloud/Enrichr/ ), a functional enrichment tool with an intuitive way of visually representing various pathway analyses (Kuleshov et al., 2016). The upregulated DEGs (*n* = 526) were submitted to Enrichr and pathway enrichment was evaluated based on the Kyoto Encyclopedia of Genes and Genomes (KEGG) and MSigDB. KEGG analysis (Okuda et al., 2008) showed that ECM-receptor interaction or PI3K-Akt signaling pathway were upregulated in the recellularized group (Figure 4C). The former pointed to stronger cell-to-ECM interactions, which is important for the tumor microenvironment; whereas the latter implied an enhancement of basic cellular process, such as proliferation or survival. MSigDB hallmark analysis (Liberzon et al., 2015) uses 50 well-defined gene sets (hallmark gene sets), summarizing representative biological states and processes. This GSEA revealed the upregulation of gene sets important for tumor behavior, such as EMT, hypoxia, and TNF-α signaling via NF-κB (Figure 4D).

### 3.5 GSEA of the *ex vivo* lung cancer model

**FIGURE 5:**
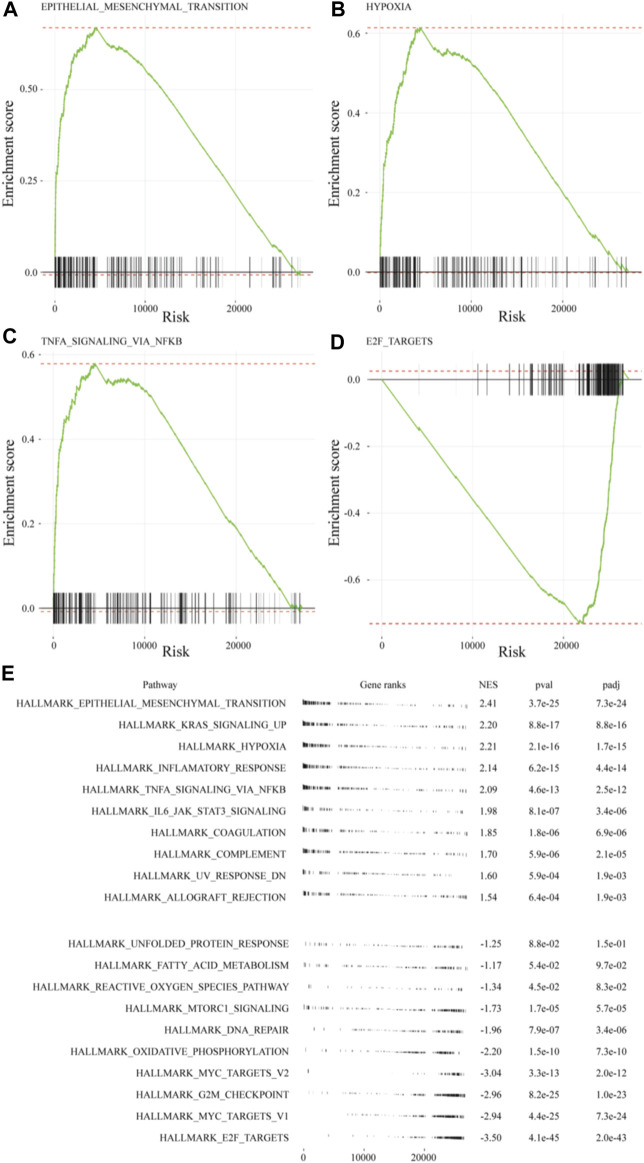
GSEA of MSigDB hallmark gene sets in A549 cells of the *ex vivo* lung cancer model. Gene sets for: **(A)** EMT (NES = 2.41), **(B)** hypoxia (NES = 2.21), **(C)** TNF-α signaling via NF-κB (NES = 2.09), and **(D)** E2F targets (NES = −3.50). **(E)** Table plot summarizing the top 10 gene sets and the bottom 10 gene sets.

The GSEA of differences in drug sensitivity between the *ex vivo* 3D lung cancer model and 2D cultures was compiled. GSEA enrichment score plots were generated for EMT, hypoxia, TNF-α signaling via NF-κB, and E2F pathway using gene sets from the MSigDB (Figures 5A–D). The green line of the enrichment score was biased to the left for highly expressed gene sets (EMT, hypoxia, and TNF-α signaling via NF-κB), and to the right for the poorly expressed E2F pathway. The GSEA normal enrichment score (NES) plots of the 50 MSigDB hallmark gene sets revealed significant differences among all 10 upregulated gene sets and seven downregulated gene sets (Figure 5E). The upregulated gene sets included EMT (NES = 2.39), hypoxia (NES = 2.19), and TNF-α signaling via NF-κB (NES = 2.07), which were also top-ranked by Enrichr. Among downregulated gene sets, the E2F pathway (NES = −3.60), which was also observed in Metascape, showed the most significant difference.

### 3.6 Drug response study using *in vitro* cultivated cells and the *ex vivo* lung cancer model

**FIGURE 6:**
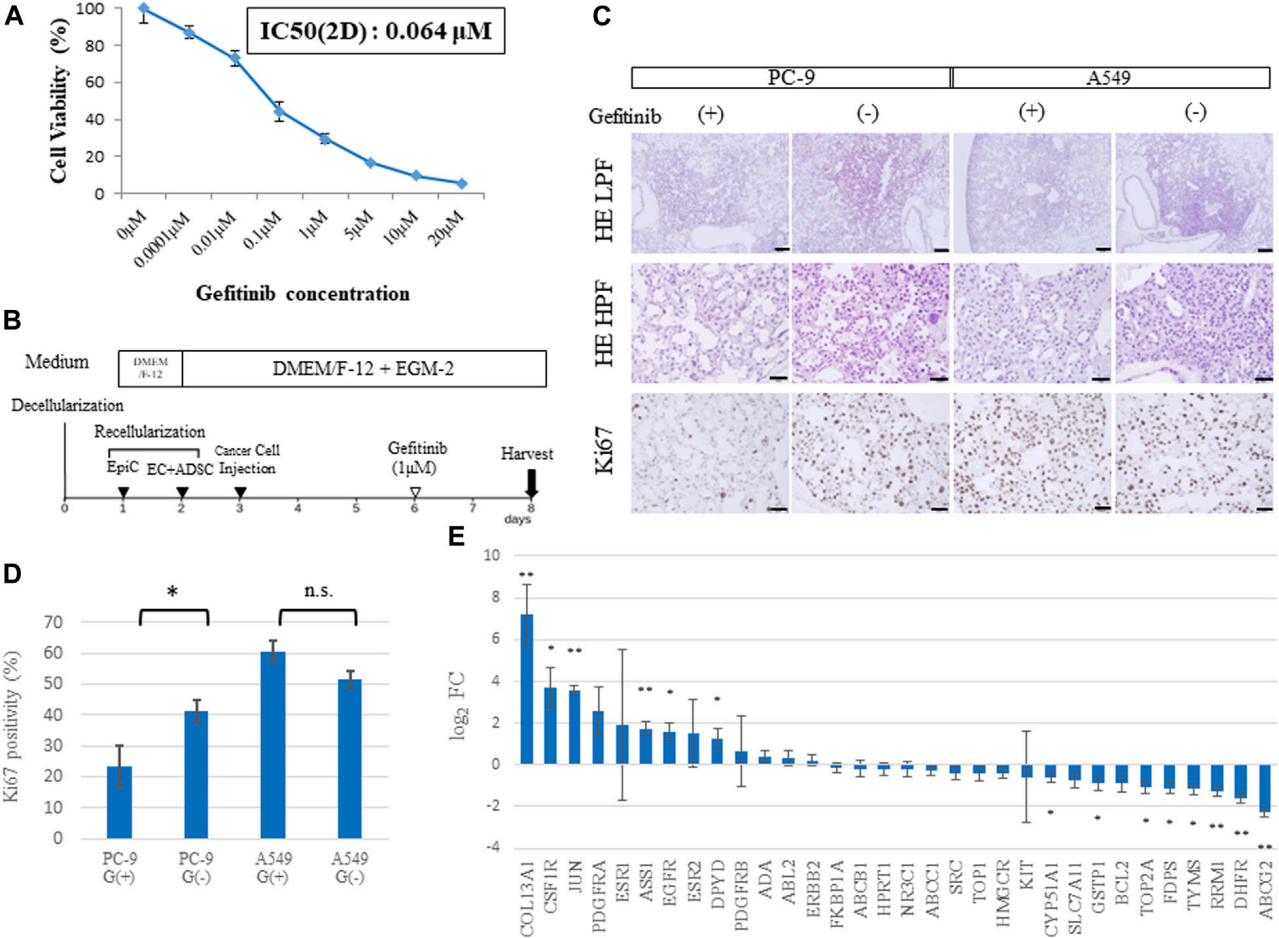
Drug sensitivity differs among *ex vivo* lung cancer models. **(A)** Cell viability assay of 2D cultured PC-9 cells treated with gefitinib at different concentrations. **(B)** Schematic protocol of the drug response study. **(C)** Histological findings from hematoxylin and eosin (HE) staining and Ki67 immunohistochemistry of EGFR-mutated PC-9 cells and EGFR wild-type A549 cells treated with gefitinib and shown at low (20×) and high (200×) magnification. **(D)** Ki67-positive cells in the samples (*n* = 3, each) stained by immunohistochemistry. **(E)** Expression of genes related to drug resistance based on RNA-sequencing data (*n* = 3, each). Scale bar; 500 μm **(A)**, 50 μm **(B–D)**. The asterisk indicates the significance of the difference (* *p <* 0.05, ** *p <* 1.0 × 10^−5^).

To assess whether the developed cancer model could help assess the efficacy of anticancer drugs, we performed a drug response study (Figure 6). The PC-9 cell line, which carries a mutated EGFR gene (exon 19 deletion), was used for *in vitro* cell viability assays against gefitinib, an EGFR-TKI. Cell viability was compared 48 h after administration of different concentrations of gefitinib (0.01, 0.1, 1, and 10 μM). The corresponding viability was 73.1%, 44.4%, 29.3%, and 9.8%, respectively (Figure 6A), and the IC_50_ was 0.064 μM. Although the IC50 of 3-D and 2-D cells are different due in part to the difference in drug delivery systems, the IC50 of 2D cells was applied and the concentration of gefitinib in the drug response test in 3D was determined to be 1 μM.

A549 cells (which carry a wild-type EGFR gene) and PC-9 cells were used to study gefitinib resistance in the 3D model. Gefitinib was mixed into the medium at a concentration of 1 μM and perfused through PA at a constant flow rate for 48 h (Figure 6B). Immunohistochemistry revealed that A549 cells in the lung cancer model presented high expression of Ki67, a proliferation marker, regardless of gefitinib treatment; whereas PC-9 cells showed a significant decrease in Ki67 expression in the group treated with gefitinib (Figure 6C). This finding was confirmed by counting Ki67-positive cells, which amounted to 23.5% vs. 41.2% (*p =* 0.04) in PC-9 cells and 60.8% vs. 51.6% (*p =* 0.06) in A549 cells with or without gefitinib treatment, respectively (Figure 6D).

Genomic elements associated with drug resistance (Wang et al., 2017) include 33 representative genes, which were examined in our *ex vivo* model (Figure 6E). A comparison of RNA sequencing results between the recellularized group and the 2D cultured group revealed that COL13A1, JUN, and ASS1 were markedly upregulated; whereas RRM1, DHFR, and ABCG2 were markedly downregulated. Furthermore, CSF1R, EGFR, DPYD, CYP51A1, GSTP1, TOP2A, FDPS, and TYMS were identified as significant DEGs. Given that JUN is involved in resistance to gefitinib (Kani et al., 2017), genetic variation in drug resistance may explain the difference between 2D cells and cancer cells *in vivo*.

## 4 Discussions

The properties of lung cancer cells in a culture dish differ in numerous respects from those of lung cancer cells that arise spontaneously *in vivo* (Griffith and Swartz, 2006; Yamada and Cukierman, 2007; Breslin and O’Driscoll, 2013). The present *ex vivo* cancer model using bioengineered lungs aimed to recreate the cancer microenvironment, including the ECM and coexistence with non-cancerous cells. Locally injected cancer cells developed tumor nodules in the bioengineered lungs, providing a clear advantage over the perfusion seeding method, which causes indiscriminate cell implantation and scattering. Histopathologically, the nodules showed various morphologies depending on the type of lung cancer cells seeded, which indicated that each cancer cell regained its own character due to the influence of the surrounding cells and matrix. Consequently, A549 cells in our model showed a characteristic glandular structure with mucin production, which could not be observed in 2D cultures. Immunohistochemical findings and Western blotting revealed upregulation of MUC-1 (Lakshmanan et al., 2015) in the bioengineered 3D model, which is consistent with previous reports of high MUC-1 expression in 3D cultures (Stratmann et al., 2014; Fecher et al., 2016). A different amount of actin was detected in the endogenous control between 3D and 2D cultures. This may be explained by the presence of various proteins in the decellularized scaffold besides lung cancer cells; whereas 2D cultures produce only the proteins produced by the lung cancer cells themselves.

Transcriptome analysis revealed various interesting differences between 2D and *ex vivo* 3D lung cancer models. The MSigDB hallmark analysis pointed to the upregulation of genes related to the EMT and HIF-1α. These genes have been widely implicated in drug sensitivity and may have a non-negligible impact on drug response testing, which is a crucial function in cancer models. During EMT, tumor cells lose their cell polarity and adhesion properties, instead acquiring the ability to migrate and invade, which not only contributes to invasion and metastasis but also to tumor recurrence and drug resistance through insensitivity to apoptosis (Iderzorig et al., 2018; Tulchinsky et al., 2019). Furthermore, EMT has been associated with the conversion to cancer stem cells (Shibue and Weinberg, 2017). HIF-1α is strongly related to tumor metastasis and angiogenesis, with elevated expression of HIF-1α being an indicator of poor prognosis (Masoud and Li, 2015; Rashid et al., 2021). HIF-1α is also involved in drug resistance in several solid tumors, including non-small cell lung cancer (Semenza, 2012). In addition, TNF-α, a central mediator of free radical-induced oxidative stress and inflammatory reactions, activates NF-κB, and this induces tumor cells to secrete various cytokines, such as VEGF, MCF-1/CCL-2, IL-8, and COX-2. These cytokines are involved in angiogenesis, cell proliferation, adhesion, invasion, and metastasis, but also cause resistance to chemotherapy (Zeng et al., 2020). Therefore, the complex interplay between these processes has a significant impact on tumor response to drugs. The downregulation of cell cycle-related genes in the *ex vivo* model points to the potential testing of drugs that act on a specific phase of the cell cycles, such as antimetabolites and microtubule polymerization/depolymerization inhibitors. The E2F pathway, which was also downregulated according to Metascape analysis, includes genes involved in the cell cycle and has been reported to affect drug sensitivity (Kurtyka et al., 2014; Hsu et al., 2019). The results of cell-to-ECM interaction and cell-to-cell crosstalk may further contribute to the sensitivity of the *ex vivo* lung cancer model compared to 2D cultured cancer cells.

Following RNA-sequencing analysis, we conducted drug tests to verify if the expression data corresponded to the actual resistance phenotype. First, cell proliferation was suppressed by the administration of EGFR-TKI to the sensitive PC-9 cell line, but not to the insensitive A549 cell line. This result proved that the model was suitable for testing anticancer drugs. Second, the effective concentration of administered EGFR-TKI was relatively high in the *ex vivo* 3D cancer model compared to 2D cultures, which could correlate with the good survival observed among cancer cells implanted in bioengineered lungs.

The present *ex vivo* cancer model has several advantages regarding existing 3D cancer models, such as lung-on-chip and organoids. First, it allows the coexistence of non-cancerous and cancer cells, whereby each cancer cell grows in its natural tissue matrix and interacts with surrounding cells to mimic a more natural cancer niche. Therefore, cultured cancer cells regain their innate character and pathological features, which cannot be observed in 2D cultures or other 3D models. Second, the methods used to analyze the model are simple and can be adapted to conventional pathological and molecular analyses, such as those used for living organs. Third, because any combination of cells can be selected, it is easy to analyze how multiple cell types, such as epithelial and endothelial cells, affect cancer cells. Fourth, mechanical stresses, such as shear stress due to circulating flow or respiratory movement, which have a significant impact on cancer cell differentiation and proliferation, can be easily applied. Fifth, this model is inexpensive and can be created in a short time (approximately 5 days). Therefore, the proposed model has strong potential for further development and applicability. With proper labeling and image analysis, it may be possible to analyze cell dynamics by time-lapse or real-time imaging. Furthermore, by adding immune cells, it will be possible to observe immune responses against both lung cancer cells and healthy alveolar cells.

There are also several limitations to this study. First, we used rat-derived cells for the regenerated lungs. To create a complete *ex vivo* model of small human lung cancer, we should use regenerated lungs that have been recellularized with human-derived cells; however, the latter does not recellularize well a rat lung scaffold. One possible reason for the difficulty of human cells to adhere may be the toxicity of residual SDS; a previous study by Obata et al. (2019) showed that when extracts from decellularized lung were mixed with culturing medium of RLMVECs, cell proliferation was promoted rather than decreased. This suggests that the growth-promoting effect of the interaction between ECM and cells is more significant than the disadvantage of residual detergent. Another possible reason may be explained by the lack of sufficient adhesion factors for human cells to innervate the decellularized rat lung scaffold due to differences in cell size and ECM composition. However, successful recellularization has been reported for human umbilical vein endothelial cells (Hashimoto et al., 2019), opening the possibility of recellularization of small human lungs with human-derived cells. In order to achieve the successful engraftment of human cells in bioengineered lungs, issues such as improvement of the protocol for human epithelial extraction, optimal conditions for culture medium and growth factors and methods of seeding epithelial cells need to be addressed and solved one by one. Another limitation comes from the use of cells derived from established cell lines, which have been selected for their adhesiveness and other characteristics that allow them to easily expand into culture dishes. Hence, their properties may differ from those of lung cancer cells harvested from actual patients encountered in clinical practice. We have succeeded in growing lung cancer cells extracted from clinical patients with lung cancer on decellularized scaffolds, but it remains to be seen whether this can be reproduced in all lung cancer cases in the future.

## 5 Conclusion

In this study, we report developing a novel *ex vivo* lung cancer model using regenerated lungs. The model is expected to the contribute to pathophysiological studies of lung cancer and the development of new anticancer drugs. Further refinement of this *ex vivo* model will advance cancer research and bridge the gap between preclinical evidence and the outcome of phase II or phase III trials.

## Data Availability

The datasets presented in this study can be found in online repositories. The names of the repository/repositories and accession number(s) can be found below: https://www.ddbj.nig.ac.jp/, DRA015799.
